# Neutrophil Gelatinase-Associated Lipocalin and Hypertensive Disorders of Pregnancy: A Cohort Study in Twin Pregnancies

**DOI:** 10.3390/jcm11144163

**Published:** 2022-07-18

**Authors:** Stephanie Springer, Marie Franz, Katharina Worda, Monika E. Gorczyca, Peter Haslinger, Christof Worda

**Affiliations:** 1 Department of Obstetrics and Gynecology, Medical University of Vienna, Spitalgasse 23, 1090 Vienna, Austria; stephanie.springer@meduniwien.ac.at (S.S.); monika.gorczyca@meduniwien.ac.at (M.E.G.); peter.haslinger@meduniwien.ac.at (P.H.); christof.worda@meduniwien.ac.at (C.W.); 2 Department of Gynecology and Obstetrics, University Hospital, Ludwig-Maximilians University Munich (LMU), 80539 Munich, Germany; marie.franz@med.uni-muenchen.de

**Keywords:** NGAL, multiple gestations, preeclampsia, hypertensive disorders of pregnancy

## Abstract

Hypertensive disorders complicate more than 10% of twin pregnancies. Several studies showed increased neutrophil gelatinase-associated lipocalin (NGAL) values in women with singleton pregnancies and preeclampsia. This study aimed to assess NGAL values in twin pregnancies complicated by hypertensive disorders. We conducted a study of 242 consecutive twin pregnancies at the Medical University of Vienna. Serum NGAL was evaluated twice during pregnancy and once in the postpartum period. Furthermore, serum NGAL values were compared between women who developed hypertensive disorders and those who had normal blood pressure. In all twin pregnancies, mean NGAL values increased significantly from the first to the second visit (*p* = 0.004) and, further, after delivery (*p* < 0.001). NGAL was significantly higher in pregnancies that developed pregnancy hypertension or preeclampsia when compared to the control group at the first visit (109.2 ± 48.9 ng/mL vs. 91.9 ± 29.4 ng/mL, *p* = 0.04, respectively). The predictive power of first visit NGAL values for development of pregnancy hypertension or preeclampsia was evaluated. When using a cut-off value of 115 ng/mL, we obtained a sensitivity of 45% with a specificity of 77%. We conclude that women with twin pregnancies who develop hypertensive disorders of pregnancy showed increased NGAL values at 11–16 weeks.

## 1. Introduction

The frequency of twin pregnancies in developed countries has increased tremendously over several decades [1,2]. Multiple pregnancies are associated with higher maternal and fetal complication rates [3].

Hypertension is a frequent medical issue, complicating up to one-tenth of pregnancies globally. Therefore, pregnancy-related hypertensive disorders represent a substantial cause of perinatal and maternal morbidity and mortality worldwide [4,5]. Numerous clinical studies postulated an increased rate of pregnancy-related hypertensive disorders in multifetal gestations [6,7,8,9,10]. Approximately 6.5% of all singleton pregnancies, 12.7% of all twin pregnancies, and 20% of all triplet pregnancies are affected by pregnancy-related hypertensive disorders [3].

Preeclampsia (PE) is characterised by elevated blood pressure values initially diagnosed after the 20th week with new-onset proteinuria, and when proteinuria is absent, by other diagnostic characteristics of typical preeclampsia-induced end-organ dysfunction, which include impaired liver function, thrombocytopenia, severe persistent epigastric pain, renal insufficiency, pulmonary oedema and new-onset of visual disturbances, headache or fetal growth restriction [5,11,12].

Although the pathogenesis of preeclampsia remains widely unidentified, the most widespread hypothesis is based on two stages: abnormal placentation as a consequence of an impaired remodelling of the spiral arteries in early pregnancy and, thus, the development of the maternal syndrome [13].

Impaired remodelling of the spiral arteries causes dysfunctional uteroplacental circulation and, as a consequence, placental perfusion worsens [14,15,16]. Placental hypoxia inside the intervillous space occurs, induces oxidative stress and increases placental apoptosis and necrosis. Ultimately, this causes endothelial dysfunction and an overstated inflammatory response [14,17]. Numerous angiogenic factors are suspected to be essential in regulating placental vasculogenesis. Soluble Fms-like tyrosine kinase-1(sFlt-1), vascular endothelial growth factor-1 and -2 (VEGF-1, VEGF-2), placental growth factor (PIGF), and sEndoglin (sENG) are important for physiologic vascular development. Serum markers of endothelial activation and dysfunction are disrupted in preeclamptic women [14,18]. The loss of regulation of vasculogenesis and endothelial control causes hypertension and increased permeability of vessels, which induces proteinuria and disrupted endothelial expression of coagulation factors, leading to coagulopathy [14,19].

Human neutrophil gelatinase-associated lipocalin (NGAL) is a member of the lipocalin protein family. It is covalently connected to neutrophil gelatinase. Major ligands for NGAL are siderophores, small iron-binding molecules, arachidonic acid, retinoids, fatty acids, prostaglandins, steroids, and matrix metalloproteinases (MMP) [20,21,22,23,24].

In pregnancy, NGAL values are significantly higher compared to those in non-pregnant women [25]. Under physiologic conditions in pregnancy, NGAL is expressed in trophoblasts, but not in the decidua [26,27]. During normal pregnancy, NGAL levels increase [28,29,30].

Several authors could show that NGAL is significantly augmented in preeclampsia and relates to disease severity [28,29,31,32,33,34,35,36,37,38,39,40,41]. Different studies have evaluated the NGAL values in singleton pregnancies and most of them reported an increase from the first to the second trimester [28,29,30,36]. Its expression increases in cell damage, inflammation, and ischemia. These facts indicate that NGAL might be connected to the pathogenesis of hypertensive disorders in pregnancy such as preeclampsia and eclampsia [34]. Recent studies described that NGAL was significantly augmented throughout the whole pregnancy (1st–3rd trimester) in those women who were burdened by preeclampsia later.

The aim of this study was to evaluate NGAL values in twin pregnancies. Furthermore, we assessed whether twin pregnancies who will develop pregnancy hypertension (PH) or preeclampsia showed values of increased NGAL at 11–16 weeks.

## 2. Materials and Methods

A total of 252 consecutive pregnant women with twins who met the inclusion criteria and were willing to participate in this prospective cohort study were included. Women were recruited at the outpatient clinic of the Department of Obstetrics and Gynaecology at the Medical University. This hospital is a tertiary care centre serving high-risk pregnancies and the annual number of deliveries was about 2500 during the study period. Of 252 women, 10 women either underwent second trimester miscarriage (without having developed PE or PH) or were lost to follow-up.

PE was defined as a blood pressure of ≥140/90 mmHg measured at least two times at least four hours apart after 20 weeks of gestation in women with a previously normal blood pressure, or a blood pressure of ≥160/110 confirmed within a shorter time interval (minutes), and proteinuria. Proteinuria was defined as ≥300 mg protein excretion in the 24 h urine collection, a protein/creatinine ratio of 0.3 mg/dl, or a urine dipstick reading of 2+ (used only if other quantitative methods were not available). In the absence of proteinuria, a new onset of thrombocytopenia or renal insufficiency or an impaired liver function or pulmonary oedema was diagnostic. Pregnancy hypertension (PH) was defined as blood pressure ≥140/90 mmHg in the absence of significant proteinuria after 20 weeks of gestation [5]. The study group (PH or PE) consisted of all patients with pregnancy hypertension and/or preeclampsia. None of the patients used acetylsalicylic acid.

During the study, patients had to undergo three specific visits. Blood and urine samples were collected at two visits during pregnancy and one after delivery. The first study visit was performed between 11 + 0 and 16 + 0 weeks of gestation. Registration of pregnancy and an ultrasound examination were performed. The due date and gestational age were calculated from the first day of the last menstruation and adjusted by ultrasound. Patients gave written, informed consent to participate in the study. In addition, a precise medical and obstetric history, including history of hypertension and kidney disease, was assessed in a structured case report form. At this visit, weight and height were measured and BMI was calculated. The second study visit was performed between 28 + 0 and 34 + 0 weeks of gestation. The third study visit was performed one to two days after delivery.

In addition, all patients received ultrasound examinations according to the standard operating procedure of the clinic for twin pregnancies. All patients received a first trimester screening (between 11 and 14 weeks of gestation) and an anomaly scan (between 20 and 24 weeks of gestation).

The study was approved by the Ethical Review Board of the Medical University and was performed according to the standards of the Helsinki Declaration. All women gave written, informed consent after a detailed explanation of the purpose of the study.

The serum blood samples were collected according to a standard operating procedure with VACUETTE^®^ tubes (8 mL Z Serum Sep Clot Activator, Greiner Bio-One GmbH, Kremsmünster, Austria). Samples were centrifugalised immediately at 4000 RPM for 10 min at 6 °C to remove debris. Centrifugation at 6 °C is the standard operating procedure for serum extraction of blood samples in our laboratory. The supernatants were divided into 1.0 mL aliquots in sterile centrifuge tubes and stored at −80 °C for further analysis. All samples were thawed only once and mixed sufficiently after a complete thaw. Quantitative measurement of serum NGAL levels was performed with a Human Lipocalin-2/NGAL Immunoassay Quantikine ELISA kit (R&D Systems, Inc., Minneapolis, MN, USA) according to the manufacturer’s protocol.

Relevant patient data were acquired retrospectively using the Viewpoint^®^ software (GE Healthcare, Wessling, Germany, software version 5.8.28.56). This is the basic perinatological database used at the department. Acquired data included maternal and fetal characteristics and pregnancy outcome. Additional information about laboratory test results was acquired using the AKIM (Allgemeines Krankenhaus Information Management) software (SAP/i.s.h.med, Walldorf, Germany). This is the main database used at the Medical University of Vienna.

Statistical analysis was performed with SPSS version 25 for MAC OS X (IBM SPSS Inc., Armonk, NY, USA) and reported as mean ± standard deviation for normally distributed continuous variables. A Kolmogorov–Smirnov test was used to identify non-normally distributed continuous variables. In case of continuous variables, the two groups were compared using the Student’s *t*-test or Mann–Whitney-*U* test as appropriate. Categorical variables were analysed with the Chi-square test or Fisher’s exact test. A logistic binary regression model was used to test the statistical significance of coefficients for PH or PE development. To evaluate the relationship between different variables, a multivariate generalised linear model with a linear scale response was performed. We constructed a receiver–operating characteristics curve to calculate sensitivity and specificity for PH or PE. Relative risk, sensitivity, specificity, positive, and negative predictive values with their 95% confidence intervals (95% CI) were evaluated. Differences were considered statistically significant if *p*-values ≤ 0.05.

## 3. Results

A total of 242 patients were evaluated in this analysis. Of these patients, 81 (33.5%) had monochorionic and 161 (66.5%) had dichorionic twin pregnancies. Mean NGAL levels increased significantly from the first to the second visit (95.0 ± 34.2 ng/mL vs. 101.4 ± 38.4 ng/mL, respectively; *p* < 0.01) and furthermore to the postpartum period (140.8 ± 69.9 ng/mL; *p* < 0.001). Of the 242 included women, 42 (17.4%) developed hypertensive disorders during pregnancy. Of these 42 patients, 30 developed PH and 12 developed PE. The control and study group only differed in BMI (Table 1).

NGAL was significantly higher in pregnancies that developed PH or PE when compared to the control group at the first visit (109.2 ± 48.9 ng/mL vs. 91.9 ± 29.4 ng/mL, *p* = 0.04, respectively). At the second visit, women with PH or PE and those who developed PH or PE after the second visit had higher NGAL values than controls (114.9 ± 49.3 ng/mL vs. 98.2 ± 34.8 ng/mL, *p* = 0.01), whereas there was no difference in NGAL values at the postpartum visit (controls: 140.7 ± 66.9 vs. PH or PE: 141.0 ± 83.7, *p* = 0.98).

The NGAL values were even significantly higher in the group of patients who developed PE only when compared to the healthy control group at the first study visit (122.5 ± 58.0 ng/mL versus 92.8 ± 30.7 ng/mL, *p* = 0.001). At the second visit, women with PE only or those who developed PE had even higher NGAL values than controls (124.6 ± 52.0 ng/mL versus 99.6 ± 36.7 ng/mL, *p* = 0.01). There was no difference at the postpartum visit (166.6 ± 85.4 ng/mL versus 138.7 ± 68.4 ng/mL, *p* = 0.15).

**Figure 1:**
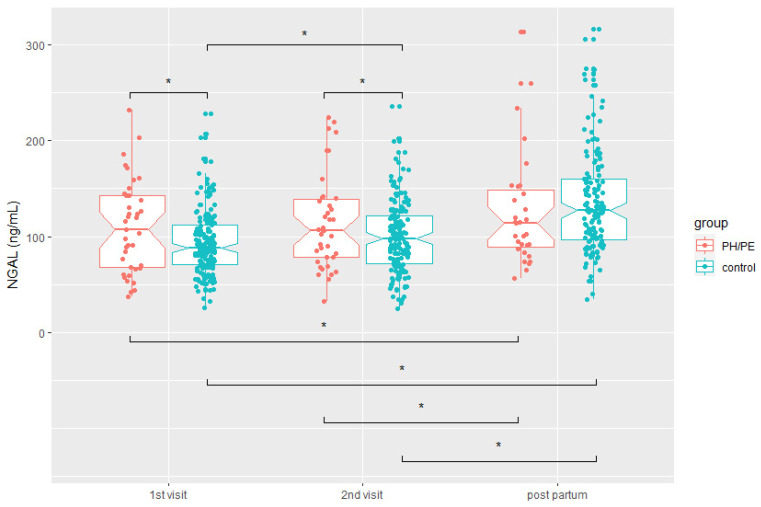
Neutrophil gelatinase-associated lipocalin (NGAL) values at different time points during pregnancy between control (*n* = 200) and study group (*n* = 42). Median, 95% confidence interval and quartiles are shown. PH, pregnancy hypertension; PE, preeclampsia; ng/mL, nanograms/millilitre. * significant difference (*p* ≤ 0.05, by Mann–Whitney-*U* test).

A comparison between the first and the second visit showed a significant increase in NGAL values in the control group (91.9 ± 29.4 ng/mL versus 98.2 ± 34.8 ng/mL; *p* < 0.001) and a non-significant increase in the PH or PE group (109.2 ± 48.9 ng/mL versus 114.9 ± 49.3 ng/mL, *p* = 0.68). In both groups, there was a significant elevation in NGAL values between the second visit and the postpartum visit (control: 98.2 ± 34.8 vs. 140.7 ± 66.9, *p* < 0.001; PH or PE: 114.9 ± 49.3 vs. 141.0 ± 83.7, *p* = 0.05) (Figure 1).

To evaluate the relationship between NGAL values at the first visit and different demographic variables (women’s age, BMI, smoking habits, chorionicity, pre-existing hypertension, ART), a multivariate regression analysis was performed. Only pre-existing hypertension was significantly linked to higher NGAL values at the first visit (Table 2).

**Figure 2:**
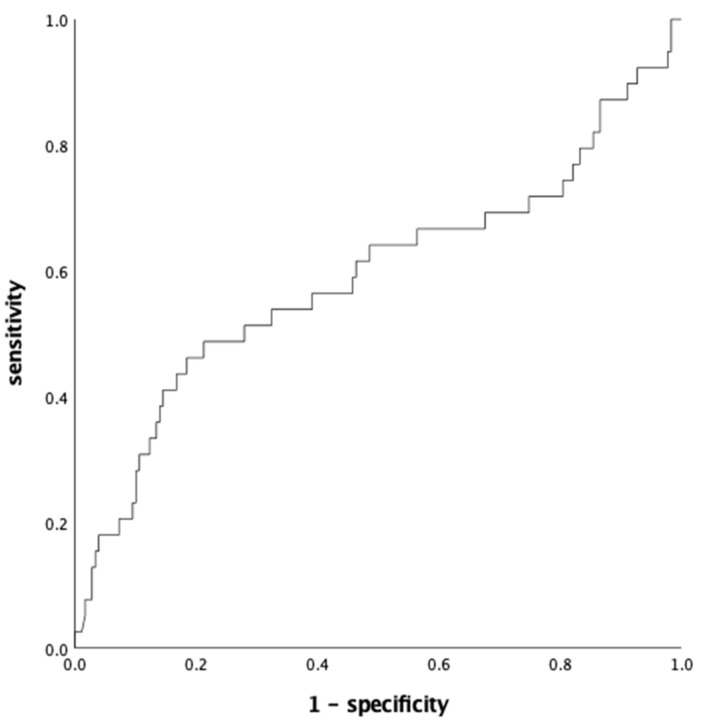
ROC curve with cut-off value of 115 ng/mL NGAL at 11–16 weeks of gestation for the detection of pregnancy hypertension and/or preeclampsia.

We have tested the predictive power of first visit NGAL values for development of PH or PE using the receiver–operating characteristics curve (ROC). ROC showed the best test characteristics when using a cut-off value of 115 ng/mL NGAL (Figure 2). With this cut-off value, we obtained a sensitivity of 45% with a specificity of 77% (area under the curve (AUC) 0.58). We calculated a relative risk (RR) of 1.5 (95% CI: 1.13–1.72). The positive predictive value (PPV) was 29% and the negative predictive value was 87%. Furthermore, we could achieve a detection rate of 45% with a false positive rate of 23%.

When using the 90th percentile for NGAL values (cut-off value 145 ng/mL) to predict PH or PE at the first study visit, the relative risk (RR) was 1.1 (95% CI: 0.96–1.34), with a sensitivity of 21% and a specificity of 93%. The positive predictive value (PPV) was 39% and the negative predictive value was 85%.

In a multivariate binary logistic regression model, the predictability of preeclampsia or pregnancy hypertension development with the use of several demographic and clinical parameters, which included NGAL (>115 ng/mL) at the first visit, was tested (Table 3). Only pre-existing hypertension and NGAL values at the first visit were significantly associated with the occurrence of preeclampsia or pregnancy hypertension in ongoing pregnancy (OR = 6.58, *p* = 0.01 and 3.83, *p* < 0.01; respectively).

## 4. Discussion

This prospective cohort study on NGAL in twin pregnancies revealed the following main findings: (i) NGAL serum levels significantly increased from the first to the second visit and reached maximum values at the time of the postpartum visit. (ii) Women who developed PH or PE had significantly higher NGAL at the first and second visit. (iii) Using NGAL values to predict the development of PH or PE at the first visit, we could observe a sensitivity of 45% and a specificity of 77%.

NGAL values have never before been reported for twin pregnancies, but several studies have evaluated the NGAL values in singleton pregnancies. Most studies reported an increase from the first to the second trimester [28,29,30,36]. Two of them observed a decrease from the second to the third trimester [30,36]. The NGAL values, which were reported from the first trimester, ranged from 12.8 ng/mL to 76.5 ng/mL [28,29,30,36,37,42]. Furthermore there were considerable fluctuations in reported NGAL values in the third trimester (15.8 ng/mL to 335.44 ng/mL) [25,28,30,31,32,34,36,39,40,41,43]. This could be attributed to methodological issues. Additionally, increased NGAL values could be reported with PH or PE [44,45]. Karampas et al. have reported an integrated model including NGAL values and maternal BMI to predict preeclampsia in singleton pregnancies with a sensitivity of 70% and a specificity of 93% [29].

Moreover, the present study is the first to report NGAL values in the immediate postpartum period. There was a significant rise between end-trimester and postpartum levels. This dynamic seems reasonable, since it has already been reported that NGAL plays a role in involution processes [44,45].

This study confirms the hypothesis that women with PH or PE revealed significantly higher NGAL values than women who do not develop PH or PE as early as in the first trimester. Nevertheless, NGAL values did not significantly rise between the first and second visit in the study group. This might be due to the high NGAL values already in early pregnancy in women who will develop hypertensive disorders later in pregnancy. The present data in twin pregnancies developing PH or PE are in accordance with the results of previous studies in singleton pregnancies. Several authors reported mean NGAL values that were about 10–18 ng/mL higher in PE patients than in controls in the first trimester [28,29,36,37]. These reported differences seem quite comparable to our results, although the initial NGAL values seemed higher in our dataset on twin pregnancies.

From a clinical point of view, early assessment of risk would be most desirable. Using NGAL (>115 ng/mL at 11–16 weeks) in twin pregnancies, we could achieve a detection rate of 45% with a false positive rate of 23%. Although a statistically significant result was observed, NGAL is not a very powerful screening test alone to predict PH or PE in twin pregnancies.

Serum markers which are routinely used to predict preeclampsia in the first trimester are Pregnancy Associated Plasma Protein-A (PAPP-A), Beta Human Chorionic Gonadotropin (Beta-HCG) and Placental Growth Factor (PlGF). Combined predictive models that include several established risk factors for PH or PE in addition to different serum markers together with ultrasound findings (e.g., uterine artery PI, ductus venosus PI) might benefit from integration of NGAL. This has to be confirmed in a separate study.

Interestingly, the multivariate regression analyses of all study visits did not show any statistical significance between the maternal BMI and NGAL values. In comparison, Cesur et al. reported significantly higher NGAL levels in obese pregnant women (110.7 ± 58.1 ng/mL vs. 42.8 ± 20.1 ng/mL, *p* < 0.001, respectively) [25]. These differences are probably due to the relatively normal BMI in our study population.

This is the first study elucidating NGAL values in twin pregnancies and preeclampsia. We investigated in our cohort study an unselected study population and followed prospectively all women throughout pregnancy and the postpartum period. There are also some limitations of our study. We did not include history of pregnancy hypertension/preeclampsia in previous pregnancies in our study and we did not incorporate uterine artery Doppler ultrasound or other serum biochemical markers such as soluble Fms-like tyrosin kinase-1 or other placental growth factors in our calculations.

## 5. Conclusions

The present study is the first study on NGAL values in twin pregnancies and its association with PH or PE. Women with twin pregnancies who developed hypertensive disorders showed increased NGAL values at 11–16 weeks of pregnancy. Many issues have to be elucidated in the future, e.g., the definition of normal ranges for NGAL in twin pregnancies and the implementation of this parameter into predictive models for the risk assessment of developing preeclampsia.

## Figures and Tables

**Table 1:** Demographic and clinical characteristics of the control and study groups.

Characteristic	Control Group (*n* = 200)	PH or PE Group (*n* = 42)	*p*-Value
Maternal age (years) *	31.6 ± 6.0	31.7 ± 6.5	0.89
GA at delivery (weeks) *	35.0 ± 4.0	35.3 ± 2.3	0.51
BMI **	24.1 (6.8)	26.3 (7.2)	0.02
Nulliparous ***	116 (58)	27 (64.3)	0.45
Smoking ***	15 (7.5)	4 (9.5)	0.70
ART ***	78 (39)	20 (47.6)	0.30
Pre-existing hypertension ***	6 (3.0)	4 (9.5)	0.05

PH, pregnancy hypertension; PE, preeclampsia; GA, gestational age; BMI, body mass index; ART, artificial reproductive technology; numbers are given as * mean ± SD and the *t*-test for statistical significance or ** numbers (percent) and the Chi-square test or Fisher’s exact test for statistical significance; *** median (interquartile range) and Mann–Whitney-*U* test for statistical significance.

**Table 2:** Multivariate regression analysis of NGAL values at the first visit (*n* = 242).

Characteristic	Standard Error	T	*p*-Value	Regression Coefficient B	95% Confidence Interval B	
Maternal age	0.45	−0.15	0.88	−0.06	−0.94	0.81
Smoking	8.71	1.67	0.09	14.52	−2.64	31.68
Dichorionic chorionicity	5.89	−0.20	0.84	−1.19	−12.81	10.42
Pre-existing hypertension	12.79	−2.11	0.03	−27.01	−52.23	−1.80
BMI	0.15	−0.16	0.87	−0.02	−0.32	0.27
ART	5.96	−0.06	0.95	−0.34	−12.08	11.41

NGAL, neutrophil gelatinase-associated lipocalin; BMI, body mass index; ART, assisted reproductive technology.

**Table 3:** Predictive model for the development of PH or PE: binary logistic regression model (*n* = 242).

Characteristic	Multivariate Analysis					
	Standard Error	Wald-Chi-Quadrat	*p*-Value	OR	95% Confidence Interval OR	
Maternal age (years)	0.032	0.085	0.321	1.032	0.969	1.100
BMI	0.011	0.006	0.937	0.999	0.978	1.021
Nulliparous	0.399	1.095	0.295	1.518	0.694	3.320
Smoking	0.561	3.162	0.075	2.712	0.903	8.141
Pre-existing hypertension	0.735	6.568	0.01	6.577	1.557	27.773
NGAL value 1st visit (>115 ng/mL)	0.39	11.87	0.001	3.83	1.79	8.24

PH, pregnancy hypertension; PE, preeclampsia; BMI, body mass index; NGAL, neutrophil gelatinase-associated lipocalin; OR, odds ratio.

## Data Availability

The data presented in this study are available on request from the corresponding author.
